# Quantifying the effect of population mixing on childhood leukaemia risk: the Seascale cluster

**DOI:** 10.1038/sj.bjc.6690664

**Published:** 1999-09

**Authors:** H O Dickinson, L Parker

**Affiliations:** North of England Children's Cancer Research Unit Department of Child Health, University of Newcastle, Queen Victoria Road Newcastle upon Tyne, , NE1 4LP, UK

**Keywords:** retrospective cohort study, childhood leukaemia, non-Hodgkin's lymphoma, population mixing, Seascale, epidemiology

## Abstract

A statistical model was developed based on Poisson regression of incidence of childhood leukaemia and non-Hodgkin’s lymphoma (NHL) in relation to population mixing among all 119 539 children born 1969–1989 to mothers living in Cumbria, north-west England, (excluding Seascale). This model was used to predict the number of cases in Seascale (the village adjacent to the Sellafield nuclear installation) children, born 1950–1989 and diagnosed before 1993. After allowing for age, the incidence of acute lymphoblastic leukaemia (ALL) and NHL was significantly higher among children born in areas with the highest levels of population mixing, relative risk (RR) = 11.7 (95% confidence interval (CI) 3.2–43) and was highest among children of incomers. The model predicted up to 3.0 (95% CI 1.3–6.0) cases of ALL/NHL in children born in Seascale compared to six observed and 2.0 (95% CI 1.0–3.4) cases in children resident, but not born, in Seascale compared to two observed. Population mixing is a significant risk factor for ALL/NHL, especially in young children, accounting for over 50% of cases in Cumbria and most cases in Seascale. © 1999 Cancer Research Campaign


*British Journal of Cancer* (1999) **81**(1), 144–151
© 1999 Cancer Research Campaign
Article no. bjoc.1999.0664

### Quantifying the effect of population mixing on childhood leukaemia risk: the Seascale cluster

**HO Dickinson and L Parker**

North of England Children's Cancer Research Unit, Department of Child Health, University of Newcastle, Royal Victoria Infirmary, Queen Victoria Road, Newcastle upon Tyne NE1 4LP, UK

**Summary** A statistical model was developed based on Poisson regression of incidence of childhood leukaemia and non-Hodgkin's lymphoma (NHL) in relation to population mixing among all 119 539 children born 1969–1989 to mothers living in Cumbria, north-west England, (excluding Seascale). This model was used to predict the number of cases in Seascale (the village adjacent to the Sellafield nuclear installation) children, born 1950–1989 and diagnosed before 1993. After allowing for age, the incidence of acute lymphoblastic leukaemia (ALL) and NHL was significantly higher among children born in areas with the highest levels of population mixing, relative risk (RR) = 11.7 (95% confidence interval (CI) 3.2–43) and was highest among children of incomers. The model predicted up to 3.0 (95% CI 1.3–6.0) cases of ALL/NHL in children born in Seascale compared to six observed and 2.0 (95% CI 1.0–3.4) cases in children resident, but not born, in Seascale compared to two observed. Population mixing is a significant risk factor for ALL/NHL, especially in young children, accounting for over 50% of cases in Cumbria and most cases in Seascale.

**Keywords**: retrospective cohort study; childhood leukaemia; non-Hodgkin's lymphoma; population mixing; Seascale; epidemiology

Since its discovery by Yorkshire television (Cutler, 1983), the childhood leukaemia and lymphoma cluster in the village of Seascale, next to the Sellafield nuclear complex in Cumbria, north-west England, has been the subject of intense public interest and extensive scientific investigation (COMARE, 1996). Initial concern that the excess could be linked to discharges of radioactive material from Sellafield was not borne out by detailed radiological studies (Black, 1984). An association between cases of leukaemia among children born in the village and doses of radiation received by their fathers while working at Sellafield before conception (Gardner et al, 1990) has not been confirmed by other studies and is not considered to be causal (Doll et al, 1994). This has resulted in attention being turned to other possible risk factors.

Kinlen has postulated that the mixing of populations from different areas, bringing together infective and susceptible individuals, can increase the incidence of childhood leukaemia and non-Hodgkin's lymphoma (NHL) and may account for the excess in Seascale and also that near the nuclear reprocessing plant at Dounreay (Kinlen, 1988, 1993; Kinlen et al, 1993). In a series of studies he has shown that marked population mixing has been associated with higher rates of childhood leukaemia and lymphoma (Kinlen, 1995; Kinlen at al, 1995). In support of this hypothesis, other research groups have also reported higher rates of childhood leukaemia in isolated, rural areas (Alexander et al, 1990), in areas with an increase in the population (Langford, 1991; Rodrigues et al, 1991) and in areas of higher socioeconomic status (Draper et al, 1991; Rodrigues et al, 1991). A study based on county district of residence at diagnosis, which analysed the effect of migration, simultaneously with the effects of various indicators of socioeconomic status, found that higher migration and greater diversity of origin of the migrants were associated with a higher risk of childhood leukaemia, and concluded that these factors, although correlated with socioeconomic status, were of more fundamental importance (Stiller and Boyle, 1996). However, no study has systematically examined the risk of childhood leukaemia in small areas in relation to population mixing to determine the extent of increase that can be explained by this factor.

The objective of the present study was to quantify the relationship between population mixing and childhood leukaemia and NHL and to determine the extent to which it can account for the excess in Seascale.

*Received 16 November 1998*
*Revised 7 January 1999*
*Accepted 27 January 1999*

*Correspondence to:* HO Dickinson

### SUBJECTS AND METHODS

The incidence of (i) leukaemia and NHL and (ii) solid tumours, for which there has been no suggestion of an association with population mixing, in children aged 1–14 years and born in Cumbria, excluding Seascale, during 1969–1989, was analysed in relation to both community and individual risk factors. Hodgkin's disease and retinoblastoma were excluded from the category of solid tumours as there is evidence of an infective aetiology for the former (Armstrong et al, 1993) and the latter has a hereditary basis in 44% of cases (Draper et al, 1992). The resulting model of the incidence of acute lymphoblastic leukaemia (ALL) and NHL was used to predict the number of cases in Seascale children, born 1950–1989. The measures of population mixing used the place of birth of parents which was available from birth certificates only from 1969 onwards but, for Seascale children born before 1969, parents' places of birth were obtained from other sources.

### Cumbrian birth cohort

The cohort comprised all children born between 1 January 1969 and 30 September 1989 to mothers usually domiciled in Cumbria, north-west England, excluding Seascale ward. Details of the birth registrations of these children were obtained from the Office for

**Table 1** Cases and age at diagnosis, among children born in Cumbria 1969–1989, diagnosed age 1–14 years, before the end of 1992

| Diagnosis | Diagnostic group (Birch and Marsden, 1987) | Age at diagnosis | | |
|---|---|---|---|---|
| | | 1–6 years | 7–14 years | Total |
| Common ALL (cALL) | Ia | 32 | 8 | 40 |
| Definitely cALL[a] | | 30 | 8 | 38 |
| Probably cALL[b] | | 2 | 0 | 2 |
| Other ALL | | 8 (1) | 5 | 13 (1) |
| ALL, subtype unknown[c] | | 3 (1) | 1 | 4 (1) |
| ALL, Definitely not cALL[d] | | 5 | 4 | 9 |
| Other leukaemias | I b, c, d | 3 (1) | 4 (1) | 7 (2) |
| Non-Hodgkin's lymphoma (NHL) | II | 4 | 4 (1) | 8 (1) |
| Total (leukaemia and NHL) | | 47 (2) | 21 (2) | 68 (4) |
| Solid tumours | III, V–XIII | 56 (23) | 32 (12) | 88 (35) |
| Person-years at risk | | 661,417 | 587,791 | 1,249,208 |

The number of cases where diagnostic tissue was not available is indicated in brackets. [a]Expressed the cALL antigen and negative for T-cell markers. [b]Negative for T-cell markers and mature B-cell features. [c]No immunophenotypic data. [d]'Null' or T-cell ALL.

**Table 2** Community characteristics, describing each ward for each time period

| Variable | Source of data | Derived from: |
|---|---|---|
| (a) Population mixing | Cumbrian birth cohort | Proportion of parents (whose place of birth was known) who were born outside Cumbria |
| (b) Proportion of child movers | 1981 census data (for births 1969–1983)<br>1981, 1991 census data (averaged for births 1984–1989) | Proportion of childhood population (age 0–15 years) not resident at that address one year before the census |
| (c) Parental diversity | Cumbrian birth cohort | Diversity of counties of origin of non-Cumbrian born parents (whose place of birth was known) (Stiller and Boyle, 1996; Shannon, 1948) |
| (d) Social class indicator | Cumbrian birth cohort | Proportion of fathers (whose social class was known) who were of social class I or II |
| (e) Density of births | Cumbrian birth cohort | Number of births per km$^2$ to mothers domiciled in ward |
| (f) Proportional change in number of births | Cumbrian birth cohort | Proportional change, between successive time periods, in number of births to mothers domiciled in ward |
| (g) Isolation (assumed to be the same for all time periods) | Bartholomew 1:250 000 digital map data, 1996 | 1 = built-up area; 2 = nearby towns, villages; 3 = isolated towns, villages; 4 = other (Alexander et al, 1990) |

National Statistics (ONS) and entered onto a computer database (Parker et al, 1997). The mother's address was grid-referenced and assigned to one of the 171 electoral wards used in the 1991 census. The fathers' and mothers' counties of birth (or regions of Scotland) were coded (Mason, 1986). Children were grouped into families using algorithms based on parents' names and measures of population mixing and social class in a ward were based on characteristics of parents.

### Case ascertainment

Cancer registrations for the cohort, recorded throughout the UK, were obtained from ONS, from six regional and national cancer registries and from scrutiny of all relevant death registrations, also obtained from ONS (Parker et al, 1997) (Table 1). Each case of cancer was reviewed centrally at the Royal Victoria Infirmary, Newcastle upon Tyne, or by the Northern Region Young Person's Malignant Disease Registry or Manchester Children's Tumour Registry, including examination of diagnostic tissue when available, case records and immunophenotypic data on leukaemias.

Prior to analysis, it was decided to consider (i) all cases of leukaemia and NHL (leukaemia/NHL); (ii) the following subgroups: common ALL (cALL), other ALL, other leukaemias, NHL; and (iii) solid tumours.

### Follow-up

There is evidence that many leukaemias diagnosed under the age of 1 year are different at a molecular level from other childhood leukaemias, possibly reflecting a different aetiology (Greaves, 1996, 1997) and so these were excluded. Each child was therefore followed up from age 1 year until he or she reached 15 years, died or emigrated, or until the end of 1992, whichever was the earliest. Hence the person-years at risk were calculated (Table 1).

### Explanatory variables

At the individual level, a measure of population mixing was derived from the place of birth of the child's parents: each child was categorized as having either both, one or neither parent born

*© Cancer Research Campaign 1999* 

outside Cumbria. Additional individual characteristics considered were: time period of birth (1969–1973, 1974–1978, 1979–1983, 1984–1989), age (1–6, 7–14 years) and social class of the child's father (ascertained from his occupation as recorded on the birth registration (Parker et al, 1997)). Social class was unknown for one case of leukaemia/NHL and 2.5% of total person-years; the place of birth of one or both parents was unknown for three cases and 6.4% of total person-years.

Community characteristics for each ward for each 5-year period were calculated from the characteristics of the parents of the babies born there (parents having more than one child born in a ward in a 5-year period contributing only once) and also from census data and maps (Table 2). This included three ward-based measures of population mixing: (a) the proportion of parents born outside Cumbria, (b) the proportion of children who had moved in the year before a census and (c) a measure of the diversity of counties of origin of the parents (Shannon, 1948; Stiller and Boyle, 1996). Four other community characteristics, (d)–(g) of Table 2, were also considered. To facilitate comparisons of the effects of the different community characteristics, the continuous variables (a)–(f) were standardized to have a range from 0 to 1.

### Model of distribution of cases

A Poisson regression analysis using maximum likelihood methods (McCullagh and Nelder, 1989) was undertaken within the statistical package Stata™ to investigate the relationship between the incidence of cancer in the predefined groups and the possible risk factors. The best model was determined by a forward step-wise regression with a backwards step at each stage (Efroymson, 1960), the significance of the improved goodness-of-fit being tested by the likelihood ratio statistic (lrs). Rate ratios (RR) are presented comparing the incidence rates in an exposed and a baseline group for the categorical variables or for trend across the range 0–1 of the standardized continuous variables. Adjacent categories of explanatory variables were amalgamated if this resulted in no significant change in the likelihood ratio statistic. Where data on an individual were missing, that person was excluded from analysis of that variable. As there is evidence of geographical clustering of ALL (Cuzick and Hills, 1991), confidence intervals (CIs) in the final model were estimated by bootstrapping (Stine, 1990). As the residual deviance may not be distributed as $\chi^2$, especially in situations such as the present, where the event data are sparse, the goodness-of-fit of the final model was checked by simulation (Bithell et al, 1995). The proportion of cases attributable to population mixing was estimated (Greenland and Drescher, 1993).

### Prediction of expected number of Seascale cases

The place of birth of parents of children born in Seascale during 1950–1968 was ascertained from birth certificates of siblings born after 1969 (the year such details were first recorded), from employee records (Parker et al, 1997), or by searching national birth indices. The total person-years at risk in children born in Seascale between 1950 and 1989 was estimated as before. The statistical model describing the distribution of ALL/NHL in Cumbria, excluding Seascale, was then used to predict the number of cases in children born in Seascale and diagnosed anywhere in the UK before the end of 1992.

Although the study was primarily of a birth cohort, estimates were made of the number of cases expected among children resident, but not born, in Seascale. Census statistics gave the number of children resident in Seascale in 1951, 1961, 1971, 1981. By combining these with data on the length of residence in Seascale of children born there (Gardner et al, 1987; Kinlen, 1993), the number of person-years at risk among children aged 1–14 years resident, but not born, in Seascale between 1950 and 1992, was estimated. The statistical model was then used to predict the number of cases among these children.

### RESULTS

### Childhood leukaemia/NHL in Cumbria, excluding Seascale

The incidence of leukaemia/NHL was significantly higher in the younger age group ($P = 0.007$) and, after allowing for this, population mixing was associated with a significantly higher rate of leukaemia/NHL. At the individual level, when only the father or only the mother was born outside Cumbria, the rate was not significantly higher than that among children with both parents born inside Cumbria (RR = 1.0, 95% CI 0.4–2.2 and RR = 1.0; 95% CI 0.4–2.3 respectively) so these categories were amalgamated (local residents). If both parents were born outside Cumbria (incomers), there was a significantly higher rate of leukaemia/NHL in their children (RR = 2.3, 95% CI 1.4–4.0) compared with incomers. At the community level, in areas where a higher proportion of parents was born outside Cumbria, there was a significantly higher rate of leukaemia/NHL (RR = 6.8, 95% CI 1.9–24) for trend. Although there was a higher incidence of leukaemia/NHL in rural wards and those of a higher social class – areas which also tended to have a high level of population mixing – none of the other individual or community variables accounted for significant variation, except

**Table 3** Rate ratios for disease sub-groups among children born in Cumbria, 1969–1989, diagnosed age 1–14 years, before the end of 1992, in relation to population movement at the individual and community level, after allowing for age

| Diagnosis | No. of cases | Children of incomers vs children of local residents | | | Trend with community population mixing | | |
|---|---|---|---|---|---|---|---|
| | | Rate ratio | (95% confidence interval)[a] | $P$[b] | Rate ratio | (95% confidence interval)[a] | $P$[b] |
| cALL | 40 | 2.6 | (1.3–5.1) | 0.008 | 9.8 | (2.0–49) | 0.007 |
| Other ALL | 13 | 2.3 | (0.7–7.6) | 0.183 | 16.3 | (1.0–259) | 0.058 |
| NHL | 8 | 2.1 | (0.4–0.9) | 0.400 | 16.1 | (0.5–551) | 0.141 |
| Other leukaemias | 7 | 1.1 | (0.1–9.1) | 0.958 | < 0.01 | (0.0–2.2) | 0.052 |
| Solid tumours | 88 | 0.9 | (0.5–1.7) | 0.739 | 1.2 | (0.3–4.0) | 0.788 |

[a]Likelihood-based confidence interval. [b]Significance, $P$, derived from likelihood ratio statistic on 1 df.

 © Cancer Research Campaign 1999

**Table 4** Rate ratios for ALL/NHL (61 cases) in relation to the various explanatory variables

| Explanatory variable | Rate ratio | (95% confidence interval) | *P* |
|---|---|---|---|
| Age | | | 0.002[b] |
| 1–6 years | 1.0 | | |
| 7–14 years | 0.4 | (0.2–0.8) | |
| After allowing for age: | | | |
| Individual characteristics | | | |
| Time period of birth | 1.2 | (0.9–1.5) | 0.154[c] |
| Social class of child's father[a] | 0.8 | (0.6–1.2) | 0.332[c] |
| Place of birth of parents:[a] | | | 0.002[b] |
| One of both parents born inside Cumbria (local residents) | 1.0 | | |
| Both parents born outside Cumbria (incomers) | 2.5 | (1.4–4.3) | |
| Community characteristics | | | |
| Population mixing | 11.7 | (3.2–43) | <0.001[d] |
| Proportion of child movers | 1.5 | (0.2–9.0) | 0.683[d] |
| Social class indicator | 2.9 | (0.8–10) | 0.105[d] |
| Density of births | 0.3 | (0.0–1.9) | 0.173[d] |
| Proportional change in number of births | 2.3 | (0.3–20) | 0.443[d] |
| Isolation | | | 0.019[b] |
| Built-up area | 1.0 | | |
| Not a built-up area | 2.0 | (1.1–3.6) | |
| Parental diversity (after allowing for population mixing) | 0.6 | (0.1–3.3) | 0.603[d] |

Confidence intervals were likelihood-based. [a]Person-years associated with missing values were excluded. Significance, *P*, is derived from the lrs on 1 df and estimates the significance of: [b]difference between two categories; [c]trend over four categories; [d]trend with standardized continuous score (range 0–1).

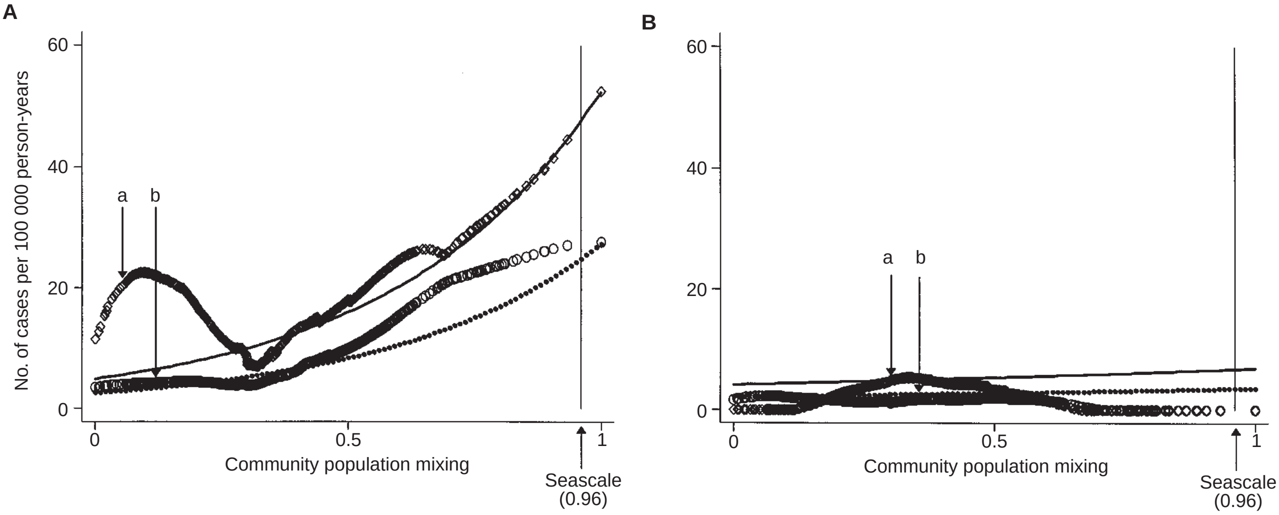

**Figure 1** Observed and predicted rates of ALL/NHL in children born in Cumbria, 1969–1989, aged (**A**) 1–6 years, (**B**) 7–14 years, diagnosed before the end of 1992. Observed rates have been Lowess smoothed (Goodall, 1990). Children of incomers: Observed (a) ◇◇◇◇◇◇◇, Expected ——; Children of local residents: Observed (b) ○○○○○○○, Expected ........

for the isolation of the ward: there was an increased risk outside built-up areas (RR = 2.1, 95% CI 1.2–3.7).

### Disease sub-group analysis

The various types of leukaemia/NHL were analysed in relation to population mixing and compared with solid tumours (Table 3). There was a substantial and significantly increased risk of cALL with increased population mixing at both the individual and community levels. There were effects of similar magnitude for other ALL (which probably included a number of unsub-typed cALL cases) and for NHL, although these effects were non-significant, probably due to the much smaller numbers of cases. These similar results for ALL and NHL suggested a commonality of effect, consistent with what is known of the overlap in their biological characteristics (Magrath, 1989). As the risk of other leukaemias did not increase with population mixing, this sub-group was excluded from the remainder of the analysis. As expected, solid tumours showed no relationship with population mixing.

Although prior decisions had been made to exclude children under 1 year of age from all analyses and to exclude cases of retinoblastoma and Hodgkin's disease from analysis of solid tumours, we checked that inclusion of these cases made little difference to the results.

© Cancer Research Campaign 1999 

**Table 5** Summary of the final multivariate model for ALL/NHL

| Explanatory variable | Estimated: Rate ratio | Estimated: (95% confidence interval) | Observed: No. of cases | Observed: Rate per 100 000 person-years |
|---|---|---|---|---|
| Age | | | | |
| 1–6 years | 1.0 | | 43 | 6.9 |
| 7–14 years | 0.9 | (0.2–3.0) | 16 | 2.9 |
| Place of birth of parents | | | | |
| One or both parents born inside Cumbria (local residents) | 1.0 | | 40 | 4.1 |
| Both parents born outside Cumbria (incomers) | 1.9 | (1.0–3.3) | 19 | 10.2 |
| Community population mixing, within the age groups: | | | | |
| 1–6 years | 10.8 | (1.7–50) | 43 | 6.9 |
| 7–14 years | 1.7 | (0.2–12) | 16 | 2.9 |

Confidence intervals were derived by bootstrapping. Person-years associated with missing values were excluded.

**Table 6** Observed and expected numbers of cases of ALL/NHL in Seascale children, born 1950–89, diagnosed age 1–14 years, before the end of 1992.

| | Age 1–6 years | | Age 7–14 years | | Total | | No of cases attributable to population mixing | |
|---|---|---|---|---|---|---|---|---|
| | No. of cases | (95% confidence interval) | No. of cases | (95% confidence interval) | No. of cases | (95% confidence interval) | No. of cases | (95% confidence interval) |
| Children born in Seascale | | | | | | | | |
| Observed | 6 | | 0 | | 6 | | n/a | |
| Expected[a] | 2.2 | (0.7–4.7) | 0.4 | (0.1–1.2) | 2.6 | (1.1–5.0) | 2.3 | (0.6–4.7) |
| Expected[b] | 2.5 | (0.8–5.4) | 0.5 | (0.1–1.2) | 3.0 | (1.3–6.0) | 2.6 | (0.8–5.7) |
| Children resident but not born in Seascale | | | | | | | | |
| Observed | 0 | | 2 | | 2 | | n/a | |
| Expected[c] | 0.8 | (0.3–1.6) | 0.2 | (0.1–0.5) | 1.0 | (0.5–2.0) | 0.8 | (0.0–1.7) |
| Expected[d] | 1.5 | (0.6–2.8) | 0.5 | (0.1–0.9) | 2.0 | (1.0–3.4) | 1.7 | (0.6–3.1) |

Confidence intervals were derived by bootstrapping. For parents of children born in Seascale, 1950–1968, unknown places of birth were (a) assumed to be inside Cumbria or (b) imputed from those known. Parents of children born outside Seascale were assumed to have been (c) born inside Cumbria or (d) born outside Cumbria.

### Childhood ALL/NHL in Cumbria, excluding Seascale

Table 4 shows the results of the analysis of ALL/NHL. Only age, population mixing and isolation of the ward were significant. After allowing for age, both individual and community level population mixing were significant after allowing for the other ($P = 0.035$ and 0.009 respectively). After allowing for these, no other variables accounted for significant variation in the rate. The effect of population mixing was greater in the younger age group (Figure 1). Although the interaction of age and community population mixing was not significant ($P = 0.24$), it was retained in the model, first because of the plausibility of an exposure during gestation or early in life preferentially affecting younger children and, second, because of prior evidence of such an effect (Kinlen, 1995; Stiller and Boyle, 1996). This final model is summarized in Table 5 and Figure 1. The proportion of cases of ALL/NHL attributable to our measures of population mixing was 0.53 (95% CI 0.20–0.73). In the younger age group, the estimated rate for children of incomers in areas with the highest levels of population mixing was 52 cases per 100 000 person-years, whereas that for children of incomers in areas with the lowest levels of population mixing was 2.5 per 100 000 person-years (Figure 1). There were five cases of ALL/NHL among children aged 1–6 years and born in the nine wards, other than Seascale, with the highest levels of population mixing (above the 95 percentile point), a rate of 32 cases per 100 000 person-years; none of these wards was close to Seascale.

### Goodness-of-fit of model

When the wards were divided into quintiles on the basis of the population mixing indicator with an approximately equal number of person-years in each quintile, the Pearson $\chi^2$ statistic comparing observed and expected numbers of cases in each category (formed by age groups within quintiles) was $\chi^2_5 = 7.6$, $P = 0.18$, indicating an acceptable fit. Second, simulation indicated that if the actual rate ratios were as estimated in Table 5, the probability of getting the observed deviance was $P = 0.39$, again indicating that the model was robust (Bithell et al, 1995).

### Observed Seascale ALL/NHL cases (Table 6)

We considered cases of ALL/NHL diagnosed up to the end of 1992 in children aged 1–14 years born between 1 January 1950 and 30 September 1989. Six relevant cases of ALL/NHL have been reported in children born in Seascale and diagnosed while living there, rates of 39 and 86 cases per 100 000 person-years in

*© Cancer Research Campaign 1999*

children aged 1–14 years and 1–6 years respectively (Black, 1984; Draper et al, 1993*a*, 1993*b*; Kinlen, 1993). Although we have ascertained details of cancers for the Cumbrian birth cohort diagnosed throughout the UK (Parker et al, 1997), no additional Seascale-born cases were found. In addition, two children born between 1950 and 1989 outside Seascale were diagnosed with ALL/NHL before the end of 1992 while resident there and aged 1–14 years (Draper et al, 1993*a*, 1993*b*; Kinlen, 1993). The place of birth of 11 of the 16 parents of these eight children was known: three had both parents born outside Cumbria and three had one parent born outside the UK.

### Predicted number of cases of ALL/NHL in children born in Seascale

A total of 1181 children were born in Seascale, 1950–1989, giving 15 199 person-years at risk. The place of birth was ascertained for 70% of parents of children born 1950–1968. Making two alternative assumptions about the unknown places of birth, the statistical model predicted the numbers of cases shown in Table 6.

### Predicted number of cases of ALL/NHL in children resident, but not born, in Seascale

The number of person-years at risk among children aged 1–14 years resident, but not born, in Seascale between 1950–92, was estimated to be 10 140 (Gardner et al, 1987; Kinlen, 1993). Assuming that this resident cohort experienced the same risks of ALL/NHL as the birth cohort and, again, making two alternative assumptions about the place of birth of the parents, the statistical model predicted the number of cases shown in Table 6.

### DISCUSSION

### Summary of results

### *Population mixing*

We have observed a significant 11.7-fold increase in the risk of ALL/NHL in children born in wards in Cumbria, excluding Seascale, with the highest level of population mixing as measured by the proportion of parents born outside Cumbria (Table 4). Within any ward, the risk is higher among the children of incomers than among children of local residents (Tables 4 and 5 and Figure 1). Thus, characteristics of both the individual and the community are important risk factors for childhood ALL/NHL. The higher rate among younger children was largely accounted for by the greater effect of population mixing in this age group. As expected, other leukaemias and solid tumours showed no variation with population mixing (Table 3), confirming that the association found for ALL/NHL is unlikely to be an artefact.

Although our measure of population mixing can only be a surrogate for the true exposure, it accounts for approximately half the cases of ALL/NHL in Cumbria. The underlying aetiological factor is likely to be an unusual pattern of exposure to infections and the risk of ALL/NHL following such exposure must be even higher and could account for the majority of cases of ALL/NHL. The differences in risk of ALL/NHL in populations of different mobility appear to be so great that any investigation of the aetiology of these diseases in children must adjust for population mixing since its effect may entirely swamp that of any other exposures.

### *Seascale*

Seascale parents were an unusually mobile group, especially in the 1950s and 1960s when there was a great influx of families into the village, most of whom had at least one parent working at Sellafield, which began operations in 1950. Over the entire time period, 1950–1989, 77% of Seascale parents for whom place of birth was known were born outside Cumbria. Thus, the children in Seascale were exposed to extremely high levels of population mixing as defined in this study.

The finding that population mixing is associated with ALL/NHL is consistent with it having a causal role in the Seascale cluster, which is predominantly of these malignancies (Draper et al, 1993*a*, 1993*b*). The final statistical model predicted 3.0 (95% CI 1.3–6.0) cases of ALL/NHL among children born in Seascale compared with six cases observed (Table 6). The place of birth of 30% of Seascale parents, 1950–1968, was estimated and this may be a source of error. However, even the most conservative estimate, assuming that all parents whose place of birth was unknown were born in Cumbria, reduced the estimate to 2.6 (95% CI 1.1–5.0) cases. The observed rate of ALL/NHL in children born in Seascale is higher than that observed in other Cumbrian wards of high population mixing, although similar high rates have been reported in areas of high population mixing elsewhere in the UK (Kinlen et al, 1997). In the early decades of operation, raw sewage was discharged from the Sellafield site into the River Ehen which flowed into the sea within a mile of Seascale contaminating the beach there, consistent with anecdotal accounts of a periodic gastrointestinal illness in Seascale (COMARE, 1996), which may have further increased the burden of infection there and consequently the risk of ALL/NHL. The model also predicted 2.0 (95% CI 1.0–3.4) cases of ALL/NHL among children born outside Seascale but diagnosed while living there, compared to two cases observed (Table 6). The confidence intervals on the predicted numbers of cases indicate that, although population mixing is likely to explain the excess of ALL/NHL in Seascale, other factors cannot be excluded.

### Comparison with other studies

The magnitude of the association found between risk of ALL/NHL and population mixing is much greater than that reported in other studies (Alexander et al, 1990; Langford, 1991; Rodrigues et al, 1991; Kinlen, 1995; Kinlen et al, 1993, Stiller and Boyle, 1996; Alexander et al, 1997). This may be because we studied the effect of population mixing in the area of residence at birth rather than diagnosis. In addition, we were able to minimize misclassification by using much smaller areal units, a ward having an average population of 2800, whereas that in previous studies ranged from 42 000 in a small census area in Hong Kong (Alexander et al, 1997) to 81 000 in a county district in England & Wales (Stiller and Boyle, 1996). We used individual records to obtain an accurate count of the population at risk in these small areas, whereas other studies have usually interpolated between widely spaced census estimates. In addition, our measures of population mixing were based on the characteristics of parents, whereas most previous studies used characteristics of the entire adult population of an area, which may be less relevant.

The associations with social class, year of birth, rural isolation and population increase were all in the direction expected from other studies (see Table 4) (Alexander et al, 1990; Draper et al, 1991; Langford, 1991; Rodrigues et al, 1991; Kinlen, 1995;

© Cancer Research Campaign 1999

Kinlen et al, 1993; Stiller and Boyle, 1996; Alexander et al, 1997), but none was significant after allowing for population mixing, implying that this was the underlying risk factor. In contrast to Stiller and Boyle, we did not find a higher risk of ALL/NHL if migrant parents had a greater diversity of origins; this may be because of the small number of migrants within each Cumbrian ward (Stiller and Boyle, 1996).

The Health and Safety Executive, in a study of 32 cases of leukaemia and NHL and 179 control children of Sellafield fathers, constructed a community migration index based on birth places of fathers but, after excluding Seascale, the correlation between this index and the ratio of observed to expected cases was not significant ($P = 0.44$) (Health and Safety Executive, 1993). However, because of the use of information from so few births and the omission of mother's place of birth, their measure of community population mixing was far less precise than that used in the present study, which was based on details of both parents of all 119 539 children born during 1969–1989 to mothers living in Cumbria.

The main limitations of the study were that, to estimate the number of Seascale cases, we extrapolated backwards in time and did not know the place of birth of all Seascale parents in early years. However, the incidence of ALL/NHL did not vary significantly over time (Table 4), so the application of the model to an adjacent time period seems reasonable.

### Possible mechanisms of the aetiology of ALL/NHL

The proportion of parents who were incomers to a ward was found to be a significant risk factor for ALL/NHL in children born there. This is consistent with Kinlen's theory of an infectious aetiology of leukaemia/NHL in which population mixing leads to an increase in contacts between susceptible and infected individuals, leukaemia/NHL being a rare and abnormal response to a possibly common infection (Kinlen, 1995). However, we also found that if parents had moved to Cumbria before their children were born, their children were at a higher risk even when community population mixing was low, implying that the risk of ALL/NHL may be associated with circumstances in prenatal life. Greaves's hypothesis (Greaves, 1997; Smith, 1997; Smith et al, 1997) concerning the aetiology of cALL suggests that a 'first hit' (a mutation in lymphocyte DNA) occurs in-utero, but our results indicate that such an effect may be acting for all ALL and also for NHL. It is possible that babies born to incomers may not receive appropriate immune protection to local infectious agents from their mothers and thus are at increased risk from infections prevalent within the community.

The risk in children with only one parent – either father or mother – born outside Cumbria was similar to that among those with two Cumbrian-born parents. A possible explanation of this is that where both parents were born outside Cumbria, they moved there together as adults; whereas when only one parent was born outside the area it is more likely that the move into Cumbria predated adulthood and so the mothers had a history of immunological stimulation similar to that of those born inside Cumbria.

### CONCLUSIONS

The study strongly supports the hypothesis that the risk of ALL/NHL, in particular in the younger age group, increases with increased exposure to population mixing during gestation or early in life. Population mixing alone could account for the Seascale leukaemia and lymphoma cluster, although the mechanism by which it causes these malignancies remains unknown. However, the possibility of additional risk factors in Seascale remains.

### ACKNOWLEDGEMENTS

We thank the following for extracting details of Cumbrian-born cancer cases: Mrs Lorna More at the North of England Young Persons' Malignant Disease Registry, Professor Jill Birch and staff at the Manchester Children's Tumour Registry, Dr Gerald Draper and staff at the National Registry of Childhood Tumours, Dr Tom Sorahan and Dr Estelle Gilman at the University of Birmingham, Mr John Stephenson and Mrs Carol McCarthy at the Northern Regional Cancer Bureau and Dr Tony Moran of the North West Regional Cancer Bureau.

We thank the United Kingdom Coordinating Committee on Cancer Research, Westlakes Research Institute and the North of England Children's Cancer Research Fund, which funded the project.

We are grateful to: Professor Leo Kinlen and Dr John Bithell for discussions and suggestions, Professor Alan Craft, Sir Richard Doll, Dr Richard Wakeford, Dr Mike Reid and Mr Mark Pearce for comments on the manuscript. We thank the Office of Population Censuses and Surveys for providing us with birth registrations, Mr David Harris and his staff at the National Health Service Central Register for providing us with cancer, death and embarkation data, Dr Mike Reid of the Royal Victoria Infirmary, Newcastle upon Tyne for reviewing cases, Mr Martin Charlton of the Department of Geography, University of Newcastle upon Tyne for areal data, Mr Tony Brady of Public Health Laboratory Service Statistics Unit for a computer program for the attributable fraction, Mr Trevor Dummer of the North of England Children's Cancer Research Unit, University of Newcastle upon Tyne, for extraction of census data and estimation of the isolation indicator, Mr Julian Smith of the North of England Children's Cancer Research Unit, University of Newcastle upon Tyne, for continuing support of the Cumbrian births database, staff of Westlakes Scientific Consulting, Cumbria for clerical assistance, Mrs Jane Salotti for administrative assistance and Mrs Katharine Kirton for secretarial assistance.

We thank Manchester Information Datasets and Associated Services (MIDAS), University of Manchester, for access to Digitised Ward Boundary Data Series (1991 Census) and to Office of Population Census and Surveys data from the 1981 Census and 1991 Census.

### REFERENCES

Alexander FE, Chan LC, Lam TH, Yuen P, Leung NK, Ha SY, Yuen HL, Li CK, Li CK, Lau YL and Greaves MF (1997) Clustering of childhood leukaemia in Hong Kong: association with the childhood peak and common acute lymphoblastic leukaemia and with population mixing. *Br J Cancer* **75**: 457–463

Alexander FE, Ricketts TJ, McKinney PA and Cartwright RA (1990) Community lifestyle characteristics and risk of acute lymphoblastic leukaemia in children. *Lancet* **336**: 1461–1465

Armstrong AA, Alexander FE, Pinto Paes R, Morad NA, Gallagher A, Krajewski AS, Jones DB, Angus B, Adams J, Cartwright RA, Onions DE and Jarrett RF (1993) Association of Epstein–Barr virus with pediatric Hodgkin's disease. *Am J Pathol* **142**: 1683–1688

Birch JM and Marsden HB (1987) A classification scheme for childhood cancer. *Int J Cancer* **40**: 620–624

Bithell JF, Dutton SJ, Neary NM and Vincent TJ (1995) Controlling for socioeconomic confounding using regression methods. *J Epid Community Health* **49**: S15–S19

© Cancer Research Campaign 1999

Black D (1984) *Investigation of the Possible Increased Incidence of Cancer in West Cumbria. Report of the Independent Advisory Group*. HMSO: London
COMARE (1996) *Committee on Medical Aspects of Radiation in the Environment, Fourth Report 1996*. Department of Health: London
Cutler J (1983) *Windscale, the Nuclear Laundry*. Yorkshire Television
Cuzick J and Hills M (1991) Clustering and clusters – Summary. In: *The Geographical Epidemiology of Childhood Leukaemia and Non Hodgkin Lymphomas in Great Britain 1966–83*, Draper G. (ed), pp. 124–126. HMSO: London
Doll R, Evans HJ and Darby SC (1994) Paternal exposure not to blame. *Nature* **367**: 678–680
Draper GJ, Vincent TJ, O'Connor CM and Stiller CA (1991) Socio-economic factors and variation in incidence rates between County Districts. In: *The Geographical Epidemiology of Childhood Leukaemia and Non Hodgkin Lymphomas in Great Britain 1966–83*. Draper G (ed), pp. 37–46. HMSO: London
Draper GJ, Sanders BM, Brownbill PA and Hawkins MM (1992) Patterns of hereditary retinoblastoma and applications to genetic counselling. *Br J Cancer* **66**: 211–219
Draper GJ, Stiller CA, Cartwright RA, Craft AW and Vincent TJ (1993*a*) Cancer in Cumbria and in the vicinity of the Sellafield nuclear installation, 1963–1990. *Br Med J* **306**: 89–94
Draper GJ, Stiller CA, Cartwright RA, Craft AW and Vincent TJ (1993*b*) Cancer in Cumbria and in the vicinity of the Sellafield nuclear installation, 1963–1990. *Br Med J* **306**: 761
Efroymson MA (1960) Multiple regression analysis. In: *Mathematical Methods for Digital Computers*. Ralston A and Wilf HS (eds), pp. 191–203. Wiley: New York
Gardner MJ, Hall AJ, Downes S and Terrell JD (1987) Follow up study of children born elsewhere but attending schools in Seascale, West Cumbria (schools cohort). *Br Med J* **295**: 822–827
Gardner MJ, Snee MP, Hall AJ, Powell CA, Downes S and Terrel JD (1990) Results of case-control study of leukaemia and lymphoma among young people near Sellafield nuclear plant in west Cumbria. *Br Med J* **300**: 423–434
Goodall C (1990) A survey of smoothing techniques. In: *Modern Methods of Data Analysis*, Fox J and Long JS (eds), pp. 126–176. Sage Publications: Newbury Park, CA
Greaves MF (1996) Infant leukaemia biology, aetiology and treatment. *Leukaemia* **10**: 372–377
Greaves MF (1997) Aetiology of acute leukaemia. *Lancet* **349**: 344–349
Greenland S and Drescher K (1993) Maximum likelihood estimation of the attributable fraction from logistic models. *Biometrics* **49**: 865–872
Health and Safety Executive (1993) *Investigation of Leukaemia and Other Cancers in the Children of Male Workers at Sellafield*. Sudbury: HSE Books
Kinlen L (1988) Evidence for an infective cause of childhood leukaemia: comparison of Scottish new town with nuclear reprocessing sites in Britain. *Lancet* **2**: 1323–1327
Kinlen LJ (1993) Can paternal preconceptional radiation account for the increase of leukaemia and non-Hodgkin's lymphoma in Seascale? *Br Med J* **306**: 1718–1721
Kinlen LJ (1995) Epidemiological evidence for an infective basis in childhood leukaemia. *Br J Cancer* **71**: 1–5
Kinlen LJ, O'Brien F, Clarke K, Balkwill A and Matthews F (1993) Rural population mixing and childhood leukaemia: effects of the North Sea oil industry in Scotland, including the area near Dounreay nuclear site. *Br Med J* **306**: 743–748
Kinlen LJ, Dickson M and Stiller CA (1995) Childhood leukaemia and non-Hodgkin's lymphoma near large rural construction sites, with a comparison with Sellafield nuclear site. *Br Med J* **310**: 763–768
Kinlen LJ, Craft AW and Parker L (1997) The excess of childhood leukaemia near Sellafield: a commentary on the fourth COMARE report. *J Radiol Prot* **17**: 63–71
Langford I (1991) Childhood leukaemia mortality and population change in England and Wales 1969–73. *Soc Sci Med (Oxford)* **33**: 435–440
Magrath IT (1989) Malignant non-Hodgkin's lymphomas. In: *Principles and Practice of Pediatric Oncology*, Pizzo PA and Poplack DG (eds), pp. 415–456. Lippincott: Philadelphia.
Mason O (1986) *Bartholomew Gazeteer of Places in Britain*. John Bartholomew & Son Ltd: Edinburgh
McCullagh P and Nelder JA (1989) *Generalised Linear Models*. Chapman and Hall: London
Parker L, Smith J, Dickinson H, Binks K, Scott L, McElvenny D, Jones S and Wakeford R (1997) The creation of a database of children of workers at a nuclear facility: an exercise in record linkage. *Appl Occupat Environ Hygiene* **12**: 40–45
Rodrigues L, Hills M, McGale P and Elliott P (1991) Socio-economic factors in relation to childhood leukaemia and non-Hodgkin lymphomas: an analysis based on small area statistics for census tracts. In: *The Geographical Epidemiology of Childhood Leukaemia and Non Hodgkin Lymphomas in Great Britain 1966–83*, Draper G (ed), pp. 47–56. HMSO: London
Shannon CE (1948) A mathematical theory of communication. *Bell System Technical Journal* **27**: 379–423
Smith M (1997) Considerations on a possible viral etiology for B-precursor acute lymphoblastic leukaemia of childhood. *J Immunother* **20**: 89–100
Smith MA, Chen T and Simon R (1997) Age-specific incidence of acute lymphoblastic leukaemia in US children: in utero initiation model. *J Nat Cancer Inst* **89**: 1542–1544
Stiller CA and Boyle PJ (1996) Effect of population mixing and socioeconomic status in England and Wales, 1979–85, on lymphoblastic leukaemia in children. *Br Med J* **313:** 1297–1300
Stine R (1990) An introduction to bootstrap methods: examples and ideas. In: *Modern Methods of Data Analysis*, Fox J and Long JS (eds), pp. 353–373. Sage Publications: Newbury Park, CA

*© Cancer Research Campaign 1999*
